# Case report: acute phosphine inhalation poisoning in a patient with Down's syndrome

**DOI:** 10.1186/s12245-026-01132-1

**Published:** 2026-02-12

**Authors:** Xingchi Yu, Jianlong Zhang, Jiangjian Xu, Keqi Dong, Lu Jin, XueBin Wen, Xiaojing Zhou

**Affiliations:** 1 Intensive Care Unit, Zhoushan Hospital, Wenzhou Medical University, Zhoushan, 316004 China; 2 Department of Biochemistry and Molecular Biology, Mudanjiang Medical University, Mudanjiang, 157011 China; 3 Department of Nursing, Zhoushan Maternal and Child Health Hospital, Zhoushan, 316004 China; 4 Department of Neurology, Zhoushan Hospital, Wenzhou Medical University, Zhoushan, 316004 China

**Keywords:** Phosphine, Down syndrome, Cardiac toxicity, Case report

## Abstract

**Background:**

Phosphine, a highly potent insecticide, can cause damage to multiple physiological systems when inhaled by humans, including the nervous, circulatory, and urinary systems. Currently, there is no effective antidote for phosphine poisoning, which highlights the importance of systematic and effective supportive treatment.

**Case presentation:**

This paper presents a case of a patient with Down syndrome who had a history of similar gas poisoning and was exposed to high-concentration phosphine gas within 10 min, resulting in inhalation poisoning and shock. Upon admission to our hospital, the patient’s left ventricular ejection fraction (LVEF) dropped to 34% (55%-75%), lactate dehydrogenase (LDH): 586 U/L (120–250 U/L), and creatine kinase-MB (CK-MB): 12.75 ng/ml (0–5 ng/ml), indicating severe cardiac function impairment. Treatment included mechanical ventilation and PICCO-guided hemodynamic management. During treatment, the patient’s whole-blood lactate concentration peaked at 12.5 mmol/L (nearly eight times the normal range), for which continuous renal replacement therapy (CRRT) was administered. Additionally, the comprehensive application of continuous norepinephrine infusion for circulatory maintenance, targeted correction of acidosis, and administration of metaraminol and dobutamine for inotropic support were crucial for improving the patient’s prognosis. Ultimately, the patient was successfully cured.

**Conclusions:**

In this case, disproportionate myocardial injury relative to pulmonary injury was observed, which may be caused by acute exposure to high concentrations of phosphine. Furthermore, the patient’s history of similar gas poisoning and Down syndrome may also contribute to this manifestation. Given the lack of a specific antidote for phosphine poisoning, our supportive treatment emphasizes the importance of PICCO for shock management and early CRRT intervention for lactate elimination and internal environment stabilization. This report provides practical experience for the treatment of phosphine poisoning, particularly for associated cardiac function damage.

## Background

Aluminum phosphide (AP) is a highly effective and toxic insecticide, commonly used for fumigating pests in granaries and grains, as well as for controlling underground rodents. AP is prone to decomposition and readily produces phosphine (PH_3_) in humid environments or under direct sunlight, which is a highly toxic gas. Common routes of human exposure mainly include direct contact with AP (primarily ingestion) and inhalation of PH_3_.

Inhalation or ingestion of phosphine allows it to enter the bloodstream, where it can directly cause failure of the cardiovascular system, liver, kidneys, and other organs, leading to multisystem dysfunction [1]. Several previous studies [2, 3]have reported that phosphine poisoning can result in acute metabolic acidosis, neuronal degeneration and plaque formation, liver and kidney failure, lung injury, and toxic myocarditis. Currently, there is no specific antidote for phosphine poisoning. Regarding the molecular mechanism of toxicity, in vitro studies have demonstrated that phosphine induces cellular damage by triggering oxidative stress. It reduces the activities of antioxidant enzymes such as superoxide dismutase (SOD) and glutathione peroxidase (GSH-Px), thereby causing cellular injury [4]. Additionally, phosphine inhibits cytochrome c oxidase, disrupts electron transport, and ultimately leads to mitochondrial dysfunction [5]. Given the nonspecific symptoms of phosphine poisoning and its high mortality rate, the development of effective supportive treatment regimens is essential.

In this case, the patient is a Down syndrome (DS) patient with a prior history of similar gas poisoning. The patient accidentally dropped approximately 100 aluminum phosphide tablets (3 g each) into water and inhaled a large amount of phosphine gas produced by decomposition in a sealed room. Ten minutes later, the patient’s family discovered the incident and immediately transported the patient to the hospital. After comprehensive supportive treatment, including respiratory support, CRRT, and PICCO hemodynamic management, the patient was successfully discharged. This case involves severe cardiac dysfunction caused by acute high-concentration PH_3_ exposure. Notably, the patient’s lungs only exhibited mild damage. We hypothesize that this may be attributed to the patient’s unique medical history and specific exposure method. The presentation of this case is conducive to the standardized management of PH_3_ inhalation poisoning and provides a practical basis for further research on the mechanism of phosphine poisoning.

## Case presentation

### Initial presentation and management

On May 12, 2025, at 13:34, a 32-year-old male was transferred to our hospital after accidentally inhaling phosphine for 4.5 h. The patient had a history of similar gas poisoning, as well as a past medical history of hepatitis B virus infection and Down syndrome. Approximately 4.5 h prior to admission, the patient inadvertently poured 100 granules (3 g each) of aluminum phosphide into water in a relatively enclosed environment, leading to the release of a significant amount of phosphine gas. After more than 10 min, family members detected the gas odor and found the patient in a confused state with oral frothing, urinary and fecal incontinence, dyspnea, vomiting, and pharyngeal discomfort. There were no limb convulsions, abdominal pain/distension, or headache. The patient was transported to a local hospital by ambulance. Initial investigations revealed: creatinine 130 µmol/L (57.0–97.0 µmol/L); white blood cell count 1.98 × 10⁹/L (3.5 × 10⁹-9.5 × 10⁹/L). Treatment included intravenous methylprednisolone, omeprazole for gastric protection, norepinephrine bitartrate for blood pressure support, furosemide for diuresis, and fluid resuscitation for internal environment correction. However, there was no significant improvement in symptoms. The patient was then transferred to our emergency department (ED) by ambulance. Upon ED admission (11:38), the patient was conscious but lethargic, with frequent vomiting of mucoid secretions, tachypnea, blood pressure maintained by norepinephrine, moist skin, mucosal injury with petechiae on the lips and oropharynx, coarse bilateral breath sounds, and a soft abdomen.

**Fig. 1:**
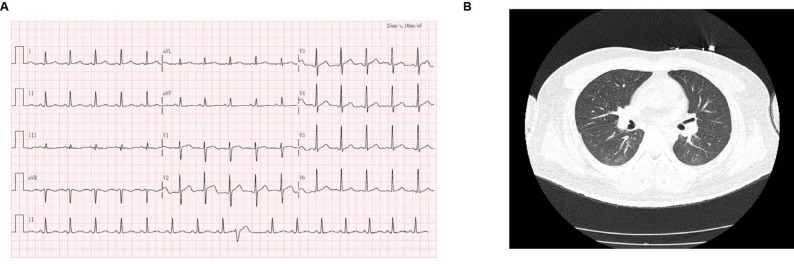
Electrocardiogram and lung CT performed on 12 May 2025, the patient has been poisoned for at least 4.5 h. (**A**) Sinus rhythm and ventricular premature beats are showed. (**B**) The bilateral lung markings exhibits a natural distribution. A few patchy hyperdense opacities are identified in both lungs. No pleural effusion or pleural thickening is observed in the thoracic cavity

Physical examination on admission was as follows: Emergency electrocardiogram (ECG) showed sinus rhythm with ventricular premature contractions (Fig. 1A) and chest CT showed mild bilateral pulmonary infiltrates (Fig. 1B). No pleural effusion or pleural thickening was observed in the thoracic cavity. At 12:35, blood gas analysis revealed: pH 7.40, partial pressure of oxygen (PO_2_) 128 mmHg, partial pressure of carbon dioxide (PCO_2_) 25 mmHg, base excess (ABE) -7.1 mmol/L, bicarbonate (HCO^3^) 15.5 mmol/L, and whole blood lactate 8.2 mmol/L. Tracheal intubation, mechanical ventilation, fluid resuscitation, acid-base correction, and supportive care were initiated, followed by admission to the intensive care unit (ICU). Physical examination upon admission to the ICU (13:54): heart rate (HR) 97 beats/min, respiratory rate 12 breaths/min, blood pressure 83/43 mmHg (maintained by norepinephrine infusion), temperature 37.2 °C. Pupils were 1.5 mm, round, equal in size, and non-reactive to light. Skin and mucosa were moist and diaphoretic, with mucosal injury and petechiae in the oral cavity and pharynx. Coarse breath sounds with scattered fine crackles were heard bilaterally. Heart rhythm was regular, the abdomen was soft, and there was no lower extremity edema. Initial laboratory tests showed the following results: Coagulation function test: plasma prothrombin time 13.6 s (10.1–13.3 s); plasma prothrombin standard ratio 1.18 (0.85–1.15); activated partial thrombin time 38.0 s (23.9–33.5 s). Blood gas analysis: serum potassium 4.90 mmol/L (3.5–5.5 mmol/L); serum sodium 134.0 mmol/L (135.0-145.0 mmol/L); chloride 115 mmol/L (96–106 mmol/L); whole blood lactate 8.2 mmol/L (0.5–1.6 mmol/L); HCO^3^ 15.5 mmol/L (21.4–27.3 mmol/L); PCO_2_ 25 mmHg (35–45 mmHg); creatinine 109.6 µmol/L (57.0–97.0 µmol/L).

The patient was immediately given medication treatment after being transferred to the ICU on 12 May, including: intensive nursing care, orotracheal intubation with mechanical ventilation, norepinephrine infusion for circulatory support, continuous infusion of sufentanil citrate and propofol for analgesia and sedation, antimicrobial therapy (piperacillin-tazobactam 4.5 g IV every 8 h), anti-inflammatory therapy (methylprednisolone sodium succinate 80 mg IV twice daily), fluid and potassium replacement for acidosis correction, stress ulcer prophylaxis (esomeprazole 40 mg IV twice daily), and comprehensive internal environment stabilization.

### Deterioration and ICU course

**Fig. 2:**
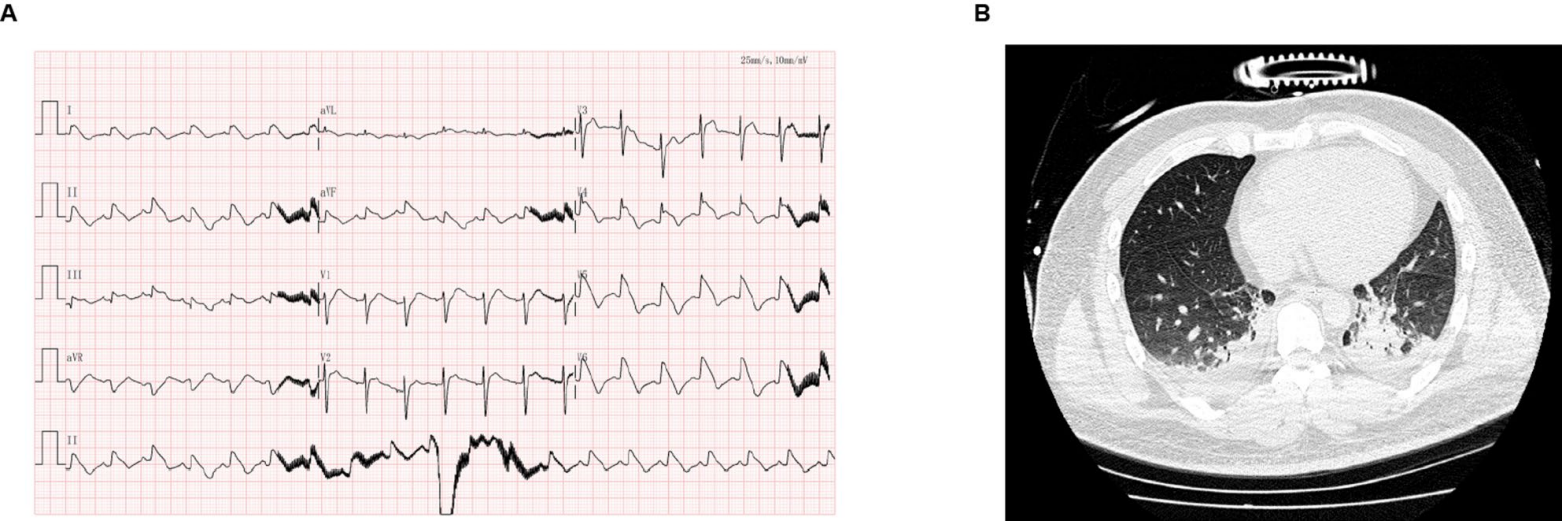
Electrocardiogram and lung CT performed on 13 May 2025, the patient’s condition has deteriorated. (**A**) Sinus tachycardia with significant ST-T segment changes. The obvious myocardial ischemia and injury are showed. (**B**) High-density shadows can be seen in both lungs, with a few exudative changes

The patient’s condition deteriorated progressively, with persistent hypotension requiring the addition of epinephrine. Echocardiography showed global left ventricular hypokinesis, reduced cardiac function, and an ejection fraction (EF) of 34%. At 13:44, ECG demonstrated sinus tachycardia with significant ST-T changes, indicating severe myocardial ischemia and injury (Fig. 2A). Meanwhile, the result of chest CT showed a few exudative changes in both lungs (Fig. 2B). By 16:00, circulation could not be maintained with norepinephrine and epinephrine. At 16:25, PICCO monitoring revealed: cardiac output (CO) 4.4 L/min (4.0–8.0 L/min); cardiac index (CI) 2.48 L/min·m^2^ (3.0–5.0 L/min·m^2^); global end-diastolic volume index (GEDVI) 285 ml/m^2^ (680–800 ml/m^2^); extravascular lung water index (EVLWI) 6.1 ml/kg (3–7 ml/kg); Pulmonary Vascular Permeability Index (PVPI) 2.3 (1.0–3.0). PICCO monitoring revealed a low cardiac index: CI 1.38 L/min·m^2^ (3.0–5.0 L/min·m^2^) on day 2, which gradually improved to 1.88 L/min·m^2^ (3.0–5.0 L/min·m^2^) by day 6 (Table 1). In addition, the Acute Physiology and Chronic Health Evaluation II (APACHE II) score was 19. Blood gas analysis showed: pH 7.08 (7.35–7.45); PO_2_ 174 mmHg (80–100 mmHg); PCO_2_ 36 mmHg (35–45 mmHg); ABE 18.6 mmol/L ( < + 3.0); HCO^3^ 10.5 mmol/L (21.4–27.3 mmol/L); PO_2_(A-a) 69.60 mmHg (< 20.0mmHg) (inspired oxygen fraction 40%); lactate concentration 12.5 mmol/L (0.5–1.6 mmol/L); anion gap (AG) 25.1 mmol/L (10.0–14.0 mmol/L); FO_2_ 427 mmHg (400–500 mmHg). These findings indicated severe internal environment derangement, high AG metabolic acidosis, and combined metabolic-respiratory acidosis. In response to elevated lactate, sodium bicarbonate was administered for acid-base correction, along with continued vasopressor support, circulatory optimization, internal environment stabilization, and ventilator parameter adjustment. A hemodialysis catheter was inserted, and continuous renal replacement therapy (CRRT) was initiated at 19:00 to clear lactate and stabilize homeostasis.

**Table 1:** Serial hemodynamic parameters from PICCO monitoring

Day	Date	CO (L/min)	CI (L/min.m^2^)	GEDVI (mL/m^2^)	EVLWI (mL/kg)	PVPI
Day 1	5.12	4.4	2.48	285	6.1	2.3
Day 2	5.13	2.44	1.38	477	7.1	2.1
Day 3	5.14	2.23	1.26	440	6.1	2.0
Day 4	5.15	3.01	1.70	647	10.2	2.3
Day 5	5.16	3.29	1.86	693	10.0	2.1
Day 6	5.17	3.33	1.88	586	10.6	2.6

CO: Cardiac Output. CI: Cardiac Index. GEDVI: Global End - Diastolic Volume Index. EVLWI: Extravascular Lung Water Index. PVPI: Pulmonary Vascular Permeability Index

Laboratory results on May 13 after CRRT treatment showed: pH 7.41 (7.35–7.45); PO_2_ 154 mmHg (80–100 mmHg); PCO_2_ 32 mmHg (35–45 mmHg); ABE − 3.2 mmol/L ( < + 3.0 mmol/L); HCO^3^ 20.2 mmol/L (21.4–27.3 mmol/L); PO_2_(A-a) 94.6 mmHg (< 20.0 mmHg); lactate concentration 3.2 mmol/L (0.5–1.6 mmol/L); AG 10.6 mmol/L (10.0–14.0 mmol/L). Partial improvement in the internal environment was observed with CRRT support, but multiorgan involvement (heart, liver, kidney, pancreas, etc.) persisted. Serial cardiac enzymes showed a dramatic rise, with CK-MB peaking at 15.82 ng/mL (0–5.0 ng/mL) and high-sensitivity troponin T (hsTnT) at 0.257 ng/mL(<0.014 ng/mL), coinciding with the nadir of cardiac function (Table 2). The patient developed refractory shock, profound cardiac dysfunction, and circulatory instability. Extracorporeal membrane oxygenation (ECMO) was considered to bridge the acute myocardial injury phase, but the ECMO team determined that vascular access was infeasible, leaving the patient at high risk for myocardial electrical storm, cardiac arrest, and death. Treatment continued with mechanical ventilation, inotropic support, corticosteroid therapy, antibiotics, and CRRT.

**Table 2:** ༎Serial cardiac biomarker profile

Date and Time	AST (U/L)	LDH (U/L)	CK (U/L)	CK-MB (ng/mL)	hsTnT (ng/mL)	MYO (ng/mL)	BNP (pg/mL)
5–12 12:04	17	169	165	2.78	0.004	103	
5–12 14:15		207	213	2.95	0.009	104	21
5–13 7:49	251	429	296	15.82	0.257	134	
5–13 14:29		586	303	12.75	0.206	73	
5–14 7:52	246	357	399	13.7	0.241	402.8	
5–15 8:17	171	382	742	5.22	0.120	340.9	217
5–16 8:12	79	345	314	1.88	0.097	184.7	203
5–17 8:05	42	422	191	1.88	0.090	113.9	194
5–18 8:08	37	483	241	1.93	0.087	290	
5–19 8:08	39	438	335	3.63	0.134	186.2	
5–20 8:01	37	458	176	3.96			
5–21 8:04	30	426	141	3.73	0.205	84.51	

AST: aspartate aminotransferase. LDH: lactate dehydrogenase. CK: creatine kinase. CK-MB: creatine kinase isoenzyme. hsTnT: High-sensitivity Troponin T. MYO: Myoglobin. BNP: B-type Natriuretic Peptide

### Recovery stage

**Fig. 3:**
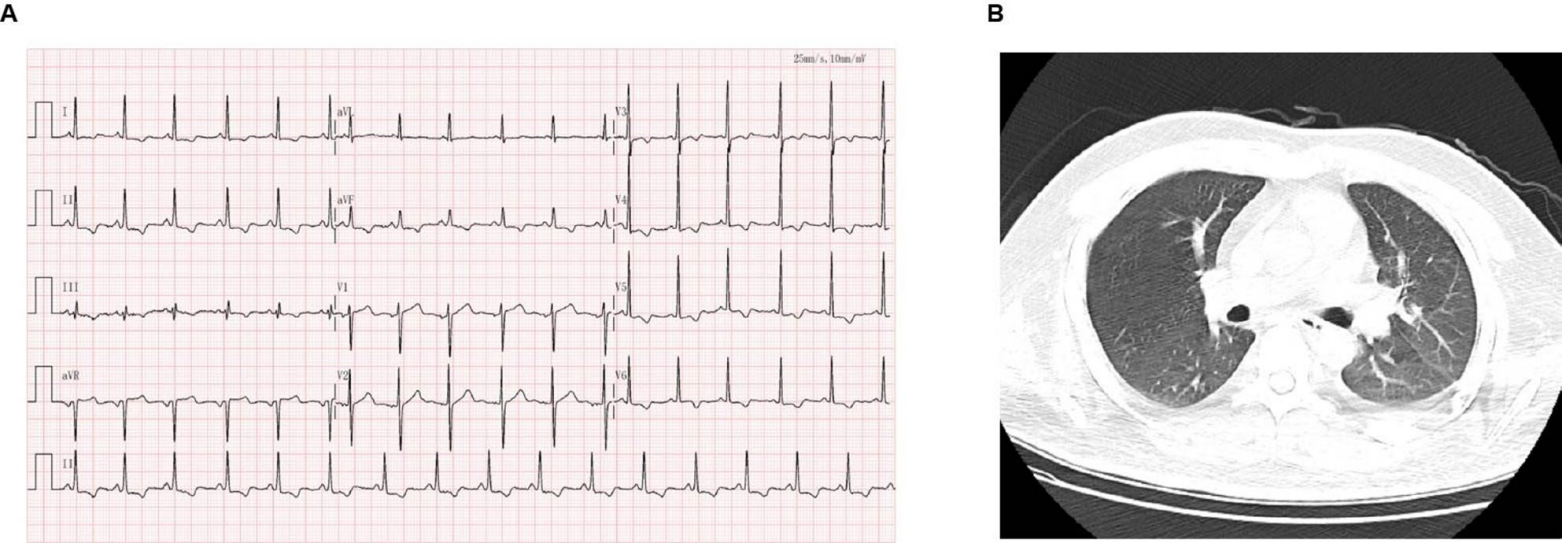
Electrocardiogram and lung CT performed on 13 May 2025. The patient’s cardiopulmonary function has improved. (**A**) Sinus rhythm. (**B**) The effusion in both lungs was significantly absorbed compared to May 13th, and there was a small amount of effusion in the left thoracic cavity

Through intensive management, cardiac function gradually improved. Bedside echocardiography on May 15 showed global left ventricular hypokinesis with an EF of 34%, and PICCO recalibration indicated slight improvement, though global hypokinesis and low cardiac output persisted, requiring ongoing norepinephrine support. Serial PICCO parameters showed progressive cardiac recovery: cardiac output (CO) 3.33 L/min and cardiac index (CI) 1.88 L/min·m^2^ on May 17. On May 19, the patient regained consciousness after sedation weaning and was successfully extubated. Echocardiography on May 20 revealed normal left ventricular systolic function (EF 59%) with reduced diastolic function. Laboratory parameters nearly normalized: creatine kinase (CK) 176 U/L (50.0-310.0 U/L); CK-MB 3.96 ng/mL (0–5.0 ng/mL); blood biochemistry showed lactate dehydrogenase (LDH) 458 U/L (120.0-250.0 U/L); aspartate aminotransferase (AST) 37 U/L (8.0–40.0 U/L). The patient was transferred to the Department of Respiratory Medicine on May 21. ECG on May 25 showed sinus rhythm, significantly improved compared with May 13 (Fig. 3A). The CT result on that day indicated a small amount of effusion in the left pleural cavity, but the amount of effusion in this patient was significantly reduced compared with the CT result on May 13 (Fig. 3B). After a comprehensive assessment, the patient’s condition was stable and they were finally discharged from the hospital successfully.

## Discussion

This paper reports a case of successful management of phosphine (PH_3_) poisoning in a patient with Down syndrome. Clinically, the patient presented with the following manifestations: oral frothing, urinary and fecal incontinence, dyspnea, vomiting, and pharyngeal discomfort. Cholinergic poisoning induced by PH_3_ was suspected, as previous cases of oral aluminum phosphide (AP) poisoning have been reported to cause cholinergic toxicity, with successful treatment achieved via atropine administration [6], this case expands on the clinical manifestations of inhalation phosphine poisoning. Additionally, the patient exhibited severe cardiac injury accompanied by disproportionately mild lung injury. A recent study reported that 12 cases of inhalational PH_3_ poisoning resulted in severe lung damage, with varying degrees of ground-glass opacities detected in all patients by computed tomography (CT); however, their liver function, renal function, and myocardial enzyme profiles remained within normal ranges [7]. Consistent with this finding, another study demonstrated that PH_3_ exposure induces lung injury in rats [8]. In the present case, the patient inhaled PH_3_ gas released by approximately 100 AP tablets (3 g each) within a 10-minute period. This discrepancy in organ injury patterns may be attributed to differences in PH_3_ exposure high concentration and duration.

Mechanistically, we hypothesize that high-concentration PH_3_ exposure preferentially damages cardiomyocytes. Although PH_3_-induced injury is considered systemic in humans, the susceptibility of different organs to PH_3_ appears to be heterogeneous, at least in this case. Previous toxicological studies on PH_3_ have shown that PH_3_ inhibits cytochrome c oxidase and generates superoxide radicals (O_2_), thereby inducing cytotoxicity [9]. As a core organ of the circulatory system, the heart may sustain more severe damage from O_2_ accumulation triggered by high-concentration PH_3_ exposure. In conclusion, the development of specific PH_3_ antidotes targeting mitochondrial proteins represents a promising therapeutic strategy for PH_3_ poisoning, which warrants further investigation.

At the individual level, relevant studies have established an association between DS and congenital cardiac malformations [10]. Although no abnormalities in cardiac development or other organ systems were detected in our patient, the potential correlation between DS and susceptibility to PH_3_ poisoning cannot be ruled out. A prior study summarized metabolism-related genes located on chromosome 21 in DS patients, including mitochondrial genes such as MRPL39 and ATP5J [11]. We postulate that the abnormal expression of DS-associated genes—particularly those involved in mitochondrial metabolism—may enhance the sensitivity of cardiomyocytes to PH_3_ in DS patients. To the best of our knowledge, no cases of PH_3_ poisoning in DS patients have been reported worldwide to date; thus, our case report holds practical significance for the management of PH_3_ poisoning in this specific population.

Exposure to PH_3_ at concentrations ranging from 550 to 830 mg/m³ for 0.5–1 h can lead to fatal outcomes [1]. Refractory cardiogenic shock is the primary cause of death in PH_3_ poisoning. Currently, no specific antidote exists for PH_3_ poisoning, and supportive treatment remains the mainstay of management, including the following interventions: PICCO hemodynamic monitoring, mechanical ventilation, enhanced circulatory support, cardiotonic therapy, and antibiotic therapy. Notably, the patient experienced a severe episode of internal environment disturbance within 24 h prior to PH_3_ exposure. Early application of continuous renal replacement therapy (CRRT) for lactate clearance and maintenance of internal environmental homeostasis may improve the poor prognosis of PH_3_ poisoning. However, limitations were observed in our supportive treatment regimen. Extracorporeal membrane oxygenation (ECMO) therapy could have facilitated the patient’s survival during the acute myocardial injury phase; nevertheless, the patient’s bilateral femoral artery diameter was less than 0.45 cm, which failed to meet the vascular puncture requirements for ECMO implantation, thereby increasing the risk of mortality. Therefore, we continued to administer CRRT for internal environment correction, norepinephrine for circulatory maintenance, and additional supportive treatments including anti-inflammatory therapy, fluid replacement, potassium supplementation, and acidosis correction. Ultimately, the patient was successfully cured, providing a novel treatment approach for the management of PH_3_ poisoning.

## Conclusion

No specific antidote currently exists for phosphine poisoning. This case underscores that acute high-concentration phosphine inhalation can cause disproportionately severe and reversible myocardial injury compared to pulmonary damage. It highlights the critical importance of aggressive, multimodal supportive care, including early hemodynamic monitoring with PICCO and CRRT, in managing such poisoning in the absence of a specific antidote.

## Data Availability

Data sharing is not applicable to this article as no datasets were generated or analyzed during the current study.
